# 1227. A Survey Study of Healthcare Resource Use, and Direct and Indirect Costs, Among Females with an Uncomplicated Urinary Tract Infection in the United States

**DOI:** 10.1093/ofid/ofab466.1419

**Published:** 2021-12-04

**Authors:** Jeffrey Thompson, Alen Marijam, Fanny S Mitrani-Gold, Jonathon Wright, Ashish V Joshi

**Affiliations:** 1 Kantar Health, New York, NY, USA, New York, New York; 2 GlaxoSmithKline plc., Collegeville, PA, USA, Collegeville, Pennsylvania; 3 GlaxoSmithKline plc, Collegeville, PA, USA, Chicago, Illinois

## Abstract

**Background:**

Uncomplicated urinary tract infections (uUTI) account for a large proportion of primary care antibiotic (AB) prescriptions. This study assessed uUTI-related healthcare resource use (HRU) and costs in US females with a self-reported uUTI.

**Methods:**

We surveyed US females aged ≥ 18 years who participated in web-based surveys (fielded August 28–September 28, 2020 by Dynata, EMI, Lucid/Federated, and Kantar Profiles). Participants had a self-reported uUTI ≤ 60 days prior, and took ≥ 1 oral AB for their uUTI. Those reporting signs of complicated UTI were excluded. HRU was measured via self-reported primary care provider (PCP), specialist, urgent care, emergency room (ER) visits, and hospitalizations. Direct costs were calculated as sum of self-reported and HRU monetized with Medical Expenditure Panel Survey estimates. Indirect costs were calculated via Work Productivity and Impairment metrics monetized with Bureau of Labor Statistics estimates. Participants were stratified by number of oral ABs prescribed (1/2/3+) and therapy appropriateness (1 AB [1^st^ line/2^nd^ line]/multiple [any line] AB) for most recent uUTI. Multivariable regression modeling was used to compare strata; 1:1 propensity score matching assessed uUTI burden vs matched population (derived from the 2020 National Health and Wellness Survey [NHWS]).

**Results:**

In total, 375 participants were eligible for this analysis. PCP visits (68.8%) were the most common HRU. Across participants, there were an average of 1.46 PCP, 0.31 obstetrician/gynecologist, 0.41 urgent care and 0.08 ER visits, and 0.01 hospitalizations for most recent uUTI (Table 1). Total mean uUTI-related direct and indirect costs were &1289 and &515, respectively (Table 1). Adjusted mean total direct costs were significantly higher (Table 2) for participants in the ‘2 AB’ cohort vs the ‘1 AB’ cohort (&2090 vs &776, p < 0.0001), and for the ‘multiple AB’ vs ‘1 AB, 1^st^ line’ cohorts (&1642 vs &875, p=0.002). Participants in the uUTI cohort reported worse absenteeism (+15.3%), presenteeism (+46.5%), overall work impairment (+52.4%), and impact on daily activities (+50.7%) vs NHWS cohort (p < 0.0001, Table 3).

Table 1. Overall mean uUTI-related healthcare resource use, direct, and indirect cost data

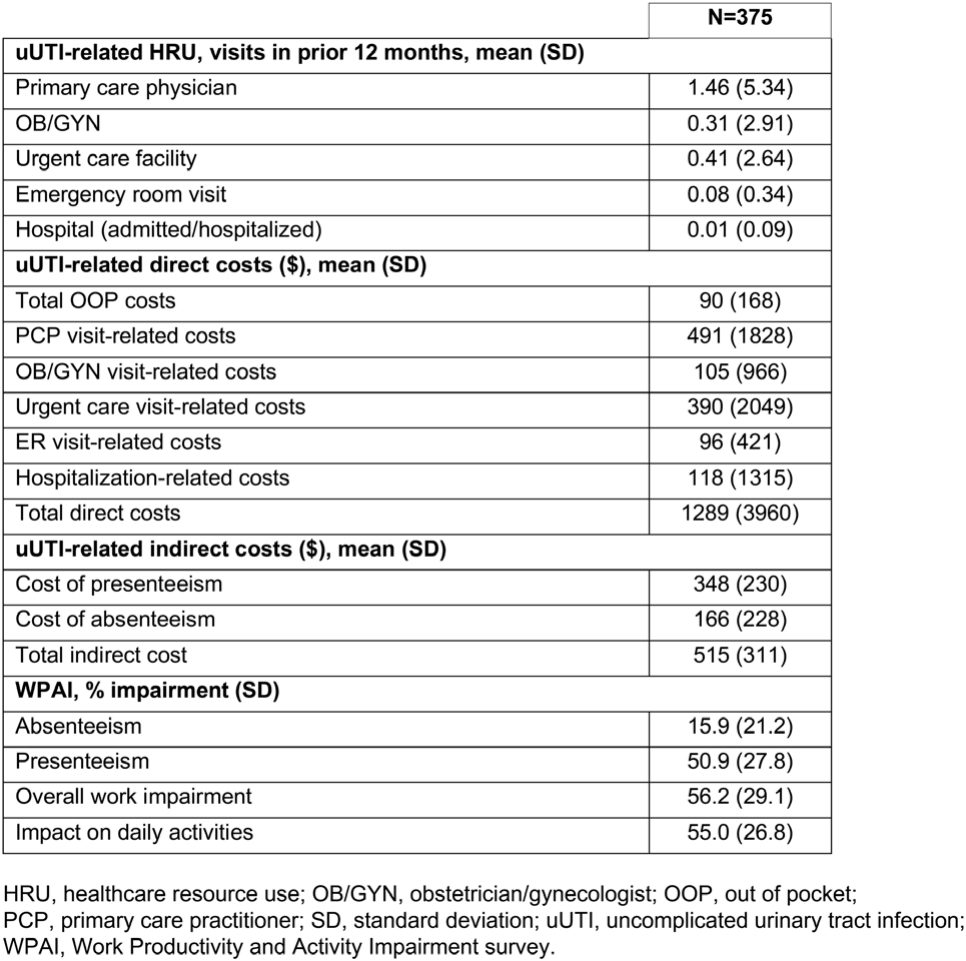

Table 2. Estimated uUTI-related direct costs stratified by (A) number of AB and (B) appropriateness of AB therapy used to treat last uUTI

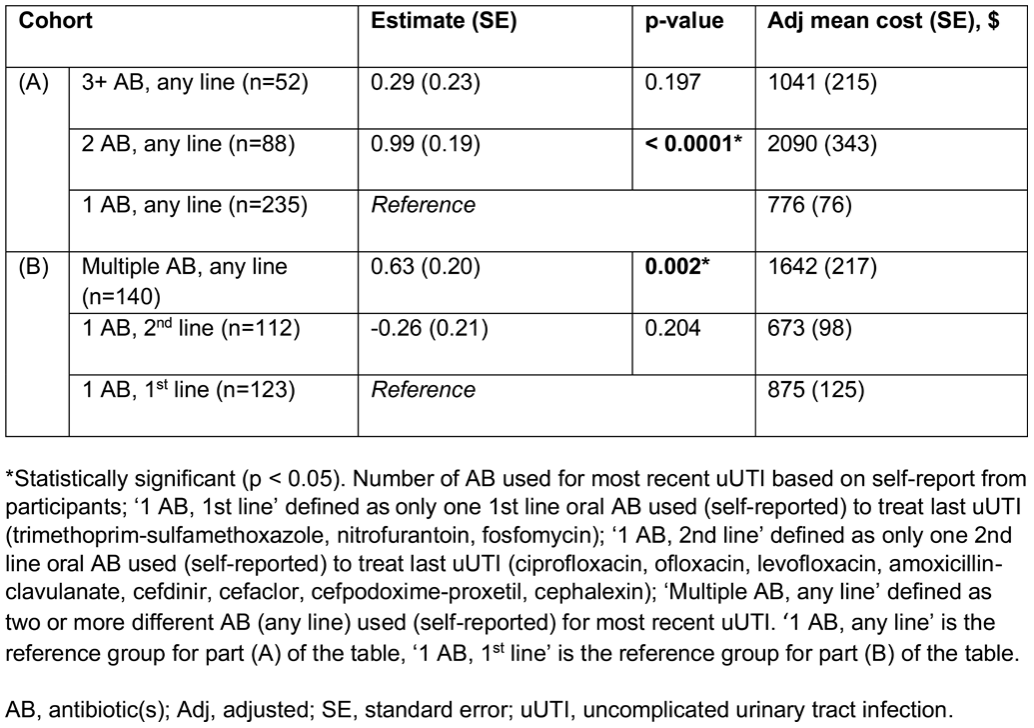

Table 3. Mean Work Productivity and Activity Impairment data for uUTI and NHWS cohorts

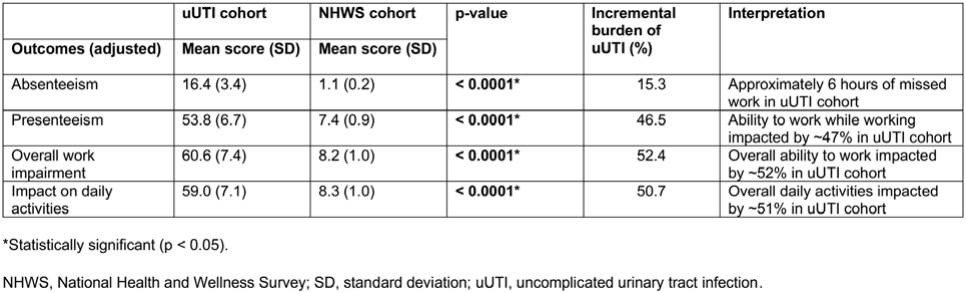

**Conclusion:**

Inadequate treatment response, evident by multiple AB use, was associated with an increase in uUTI-related costs, including productivity loss.

**Disclosures:**

**Jeffrey Thompson, PhD**, **Kantar Health** (Employee, Employee of Kantar Health, which received funding from GlaxoSmithKline plc. to conduct this study) **Alen Marijam, MSc**, **GlaxoSmithKline plc.** (Employee, Shareholder) **Fanny S. Mitrani-Gold, MPH**, **GlaxoSmithKline plc.** (Employee, Shareholder) **Jonathon Wright, BSc**, **Kantar Health** (Employee, Employee of Kantar Health, which received funding from GlaxoSmithKline plc. to conduct this study) **Ashish V. Joshi, PhD**, **GlaxoSmithKline plc.** (Employee, Shareholder)

